# Deciding for others diminishes model-based decision-making but depends on individual prosociality

**DOI:** 10.1038/s41539-025-00397-0

**Published:** 2026-01-10

**Authors:** Yangchu Huang, Xinyi Du, Shanshan Zhen

**Affiliations:** Department of Social and Behavioural Sciences, City University of Hong Kong, Hong Kong, China

**Keywords:** Mathematics and computing, Neuroscience, Psychology, Psychology

## Abstract

Acting successfully in dynamic environments requires learning supported by two systems that differ in computational demand: a fast, model-free system that repeats rewarded actions, and a more effortful model-based system that uses a mental model of the task structure to guide flexible, goal-directed decisions. A key open question is whether people engage effortful model-based strategies to the same extent when deciding for themselves versus others, and which computations underpin self-other differences. Using a two-step task with reinforcement learning drift-diffusion modelling in 92 adults, we found that deciding for others slowed down model-free learning and reduced reliance on model-based strategies, with the latter partially mediated by differences in non-decision time. Moreover, individual differences in social value orientation predicted the self-other discrepancy in model-based decision-making, with more prosocial individuals showing smaller gaps. Together, these findings identify the computational mechanisms underpinning prosocial model-based decision-making and demonstrate how individual differences modulate this computation.

## Introduction

As social creatures, people do not live in a vacuum. They frequently make decisions for others and rely on decisions made by others. Examples include parents purchasing insurance for their children, traders selecting investment strategies for their clients, and government leaders deciding public policies that impact a large population of citizens in a country. All these kinds of decisions require deliberative considerations and flexible adaptation to changing circumstances in everyday life. Such prosocial goal-directed decision-making involves the reliance on a mental model of the environment that informs action selection^1^. Reinforcement learning theory formalises this goal-directed behaviour as model-based learning^2^. This learning strategy involves understanding the structure of complex environments^3,4^, constructing a mental model representing the structure, and planning moves within that model. This process is akin to the strategic moves in a chess game, where players plan their moves based on their understanding of the game’s structure and potential responses from their opponent. In contrast, an agent’s action can also be guided by a model-free system^5^. Model-free learning relies on repeating previous choices with favourable outcomes and avoiding those leading to undesirable results. This process is similar to trial-and-error learning. For instance, after being burned by touching a hot stove, a person avoids repeating that action in the future. Prior studies have reported that individuals employ a combination of these two strategies during decision-making^6,7^. Consistent with this distinction, a quantitative fMRI meta-analysis also demonstrated that these two strategies engage overlapping (e.g., ventral striatum) yet distinct brain regions, with model-based decision-making engaging the medial prefrontal cortex and orbital frontal cortex, model-free decision-making engaging the globus pallidus and caudate^8^. While previous studies have extensively investigated model-based and model-free systems for personal decisions, far less is known about how this balance may shift when deciding on behalf of others, or how individual differences may shape these computations.

Model-based decision-making needs the construction and use of mental models to guide forward planning, which is cognitively demanding^9^. As people are usually averse to investing cognitive effort and often avoid it^10^, they tend to conduct model-based decision-making only when the potential gains are worth the cognitive effort^9,11–13^. Considering this cost-benefit analysis of engaging model-based decision-making, an important question is whether people extend this effort to others. Specifically, are individuals willing to bear the cognitive costs of model-based decision-making to enhance others’ outcomes, and what computational processes underlie this prosocial investment?

Previous studies have shown that people not only discount rewards according to physical efforts required for themselves, but also discount rewards even more sharply when deciding to exert physical efforts for others, demonstrating prosocial apathy^14,15^. This pattern of physical efforts also generalises to cognitive efforts. For example, when learning to obtain rewards for others, people exhibited slower learning speed than when learning for themselves^16^. In tasks where cognitive effort was defined as solving three-digit addition problems, individuals were less inclined to exert effort to earn rewards on behalf of a charity or a stranger than for themselves, reflecting a linear devaluation of rewards as cognitive effort increased^17^. Collectively, these findings suggest that people may be less inclined to engage in cognitively demanding, model-based decision-making for others compared to themselves. A small number of studies have directly examined model-based decision-making for others, but their results have been mixed. Lockwood et al.^18^ reported that when participants made decisions to prevent painful shocks for others, they prioritised model-free strategies over model-based strategies. In contrast, Navidi et al.^19^ observed no significant self-other differences in the involvement of model-based decision-making when participants made decisions to obtain rewards, though in their design the recipient was physically present and evaluating the participant’s performance—manipulations that may have altered participants’ motivational dynamics. Importantly, both studies inferred decision processes solely from participants’ choices, without considering response times or the latent cognitive components that contribute to prosocial model-based decision-making. The present study addresses this gap by applying a reinforcement learning drift-diffusion model (RLDDM)^20,21^ to simultaneously account for choices and response times. This approach allows us to decompose decisions into distinct computational processes, providing a more precise description of the dynamics underlying the decision process^22^.

More specifically, the RLDDM describes value-based decisions as evidence accumulation processes^21^: When deciding between two options, noisy evidence favouring one option against another is accumulated over time. Responses are made until the gathered evidence hits one of the decision boundaries corresponding to two available options (see Fig. 2a for a schematic illustration of the RLDDM). RLDDMs have been widely applied in various literature targeting mechanisms underlying decision-making^22–27^. The strength of RLDDM is its ability to identify the specific cognitive computation affected by an experimental effect of interest. For example, these models have revealed the role of dopamine neurotransmission in regulating decision thresholds^23^, the reduced decision thresholds and valuation signals associated with gambling disorder^22^, and the declined choice consistency in strategic prosocial behaviours after receiving testosterone^24^. In the context of prosocial model-based decision-making, the merit in using RLDDMs is twofold. First, RLDDMs can provide more precise and reliable estimates of whether and how people adjust their use of model-based strategies when making decisions for an anonymous other^28^. Second, RLDDMs can disentangle mixed cognitive mechanisms underlying higher decision noise when deciding on behalf of others^29,30^: the reduction in decision thresholds and the decline in integration of value differences into the rate of information uptake^22^. While both mechanisms can lead to more random choices (less choice consistency), conventional models that only incorporate choices cannot dissociate them. Thus, RLDDMs may provide novel insights into cognitive mechanisms underlying prosocial model-based decision-making.

In addition, individual differences are likely to influence the extent to which people engage in model-based decision-making for others, as prosociality and the motivation to benefit others can vary substantially across individuals^14,31–37^. For example, social value orientation (SVO), a stable personal trait reflecting how individuals assign subjective weights to others’ interests relative to their own when allocating resources^38–40^, has been linked to risky decision-making for others^41^ and neural processing of others’ financial outcomes^42^. In these studies, proselfs demonstrated less sensitivity to others’ gains and losses than prosocials. According to the social dual-process theory^43–46^, individual differences in this social preference may stem from the variability in the reliance on model-based and model-free systems. Corroborating this notion, Oguchi et al.^47^ found that proselfs relied more on the model-based system than prosocials when earning rewards for themselves. While SVO was closely linked to the model-based system in personal decisions, less is known about whether SVO can also account for prosocial model-based decision-making in contexts of interpersonal decisions. Because engaging model-based decision-making requires allocating limited cognitive resources, it is plausible that the link may generalise to social contexts: SVO may modulate the balance between model-based and model-free decision-making in self-related versus other-related decisions. Specifically, individuals with a proself orientation may show stronger model-based engagement when maximising their own rewards but reduced engagement when deciding for others. In contrast, those with a prosocial orientation may display relatively less difference between self- and other-related contexts, engaging in model-based decision-making not only for themselves but also when making decisions for others.

**Fig. 1:**
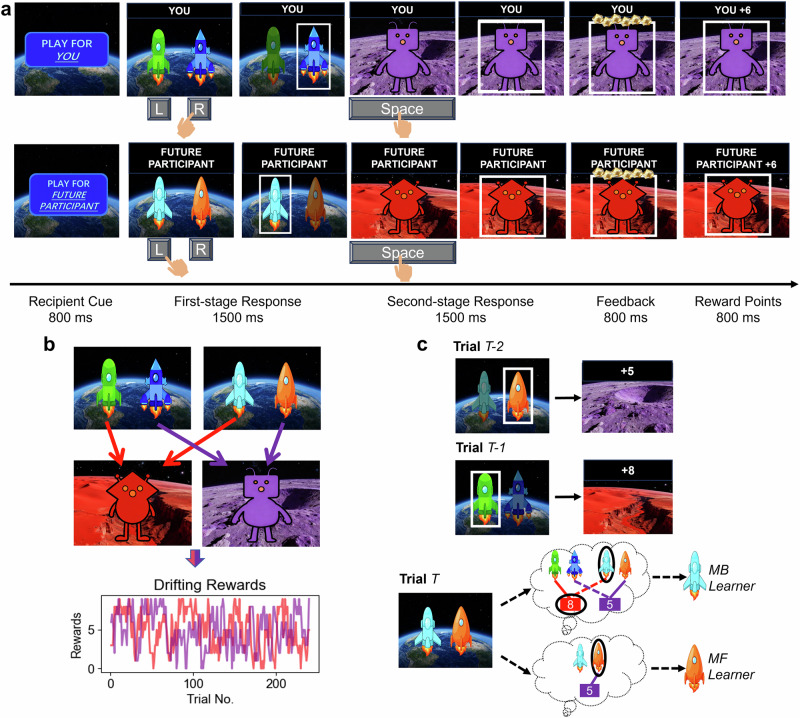
Task design and transition structure. **a** Trial structure. At the beginning of each block, the recipient was explicitly cued for 800 ms. Then the target recipient would present above the stimuli throughout the trial. Participants started randomly in one of the two first-stage states where they were instructed to select one spaceship by choosing left or right to visit the desired planet within a time limit of 1500 ms. Once participants responded, the remaining time (1500 ms - response time) would be occupied by the choice highlight. Then, participants had 1500 ms to press the ‘Space’ key during the second-stage response to obtain the reward. The aliens on each planet delivered their drifting rewards only if the keypress was successfully registered. Notably, only the first stages of the task involved decisions, the second-stage keypresses served solely to reveal the reward outcomes. Participants were asked to gain as many rewards as possible. These reward scores would be converted to real bonus money for themselves or for an anonymous future participant. **b** Task transition structure. The task contains two distinct first-stage states, each state has a pair of spaceships. Each spaceship deterministically took participants to one of the planets. The drifting rewards followed two independent Gaussian random walks (*μ* = 0, *σ* = 2), reflecting at boundaries (0 and 9). **c** An example trial sequence that distinguishes model-based from model-free strategies. Model-based strategies utilise a mental model of the task structure, allowing model-based learners to infer unexperienced transitions (represented by dashed lines) and then select the spaceship that they expect to yield better payoffs (represented by black circles), as shown in the thinking cloud of the model-based learner. On the contrary, model-free strategies simply follow previous reward history, restricted to the choices in a specific pair of spaceships, as shown in the thinking cloud of the model-free learner. Solid lines indicate experienced transitions from the previous trial history.

**Fig. 2:**
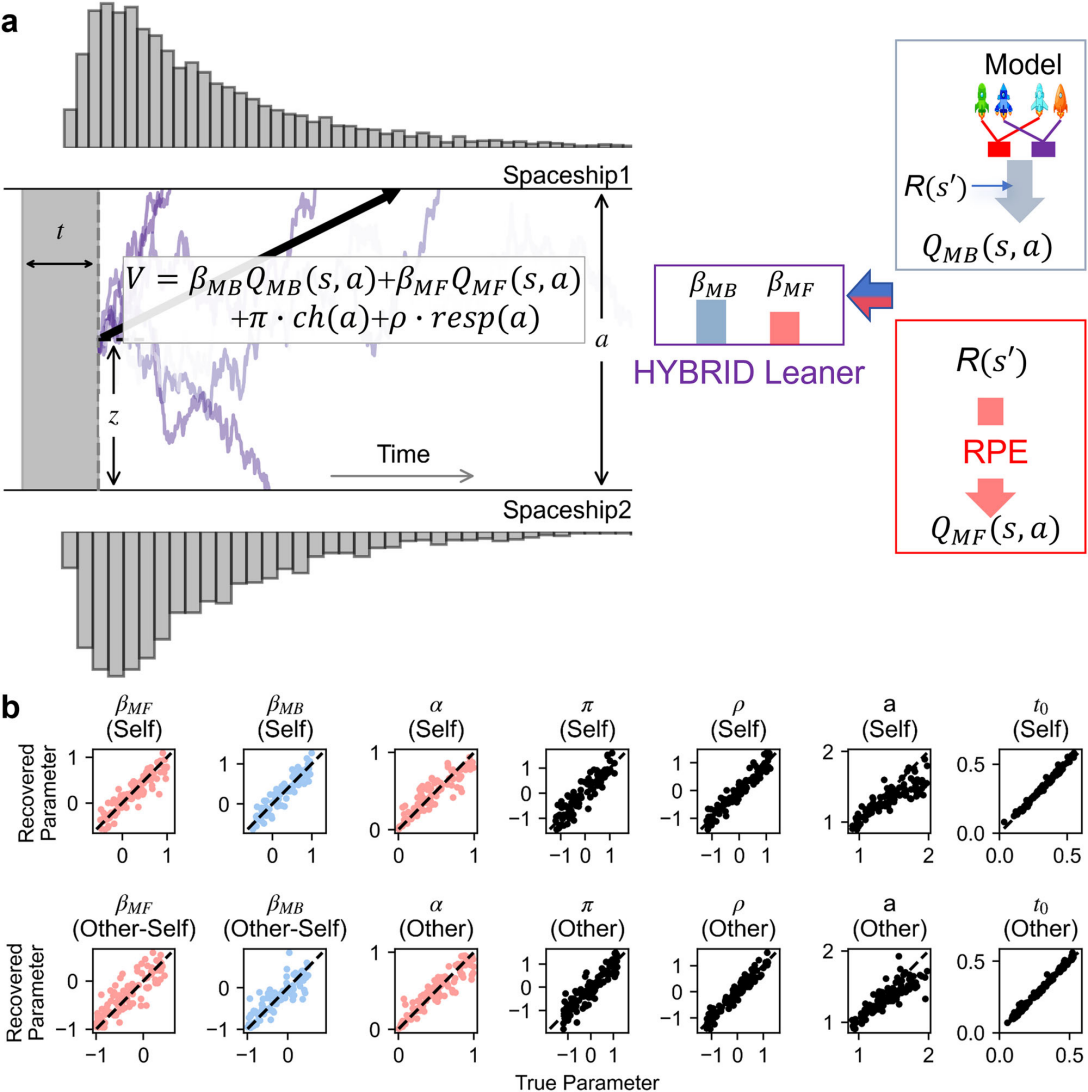
Schematic illustration of the RLDDM and parameter recovery. **a** The selection of a spaceship in the first-stage state was formalised as an evidence accumulation process, where the value difference between two spaceships drove the evidence gathering, until the noisy evidence surpassed one of the decision boundaries. The evidence gathering starts from the initial point *z* = 0.5*a, after the non-decision time *t*. The decision boundary is denoted as *a*. Four distinct cognitive mechanisms determine the value difference: model-based and model-free state-action value estimates (*Q*_*MB*_(*s*, *a*) and *Q*_*MF*_(*s*, *a*), as shown in the right panel), choice stickiness, and response stickiness. *β*_*MB*_: model-based weight; *β*_*MF*_: model-free weight; *π*: choice stickiness; *ch*(*a*): choice repetition, *ch*(*a*) = 1 if the agent repeats the previous choice, otherwise *ch*(*a*) = 0; *ρ*: response stickiness; *resp*(*a*): motor repetition, *resp*(*a*) = 1 if the agent repeats the previous keypress, otherwise *resp*(*a*) = 0. **b** Parameter recovery analysis indicated that true parameters can be well recovered using our RLDDM, suggesting this model can successfully capture self-other differences, as well as individual variability in these parameters. Red dots represent model-free components: model-free weights and model-free learning rate *α*. Blue dots represent model-based components: model-based weights. The black dashed line in each subplot represents the diagonal line where recovered model parameters equal the ground truth (simulated parameters).

In this study, our aim was to examine (1) whether deciding for others altered the involvement of model-based and model-free systems, (2) what specific cognitive computations drove the shifted decision dynamics underlying these two learning systems, and (3) whether potential self-other differences in model-based decision-making could be explained by individual differences in the SVO. To address these questions, we adapted a well-established two-step task^9,48^, where 92 participants decided between different spaceships in the first stage to visit the desired second-stage planet and then received rewards for both themselves and for an anonymous future participant in a gamified setting (Fig. 1a). In this task, each spaceship deterministically took participants to one of the two planets, where an alien would provide participants with drifting reward points (Fig. 1b). The task dissociates model-based from model-free strategies by capturing the behavioural signature that reflects the use of a mental model of the task structure: Using a mental model of task structure enables agents to consistently select the spaceship they expect will visit the higher-paying planet, as they can predict each spaceship’s destination even without recent experience of that transition (see the dashed lines in the Trial *T* shown in Fig. 1c, which represents inferred transition based on the mental model). In contrast, without a mental model, model-free learners rely solely on past reward experiences (specific state-action-outcome associations, as shown in Fig. 1c) and are less likely to arrive at the higher-paying planet due to drifting rewards and interleaved first-stage states (i.e., different pairs of spaceships). Critically, to sum up, the use of model-based strategies can lead to better payoffs than model-free strategies in this task^48^, as a mental model of the task structure allows the agent to transfer learning experiences from one pair of spaceships to another pair. This setting effectively created a trade-off between the potential gains of model-based decision-making and the putative cognitive cost associated with performing it for oneself and others. We further utilised a reinforcement learning drift-diffusion model (RLDDM, Fig. 2) to examine the self-other differences in the engagement of model-based and model-free systems, as well as to uncover the underlying cognitive computations. We also tested whether SVO can explain the potential self-other differences in model-based decision-making, with the hypothesis that prosocial individuals would demonstrate less self-other differences in model-based decision-making across two recipients.

## Results

### Model-agnostic analysis results

**Fig. 3:**
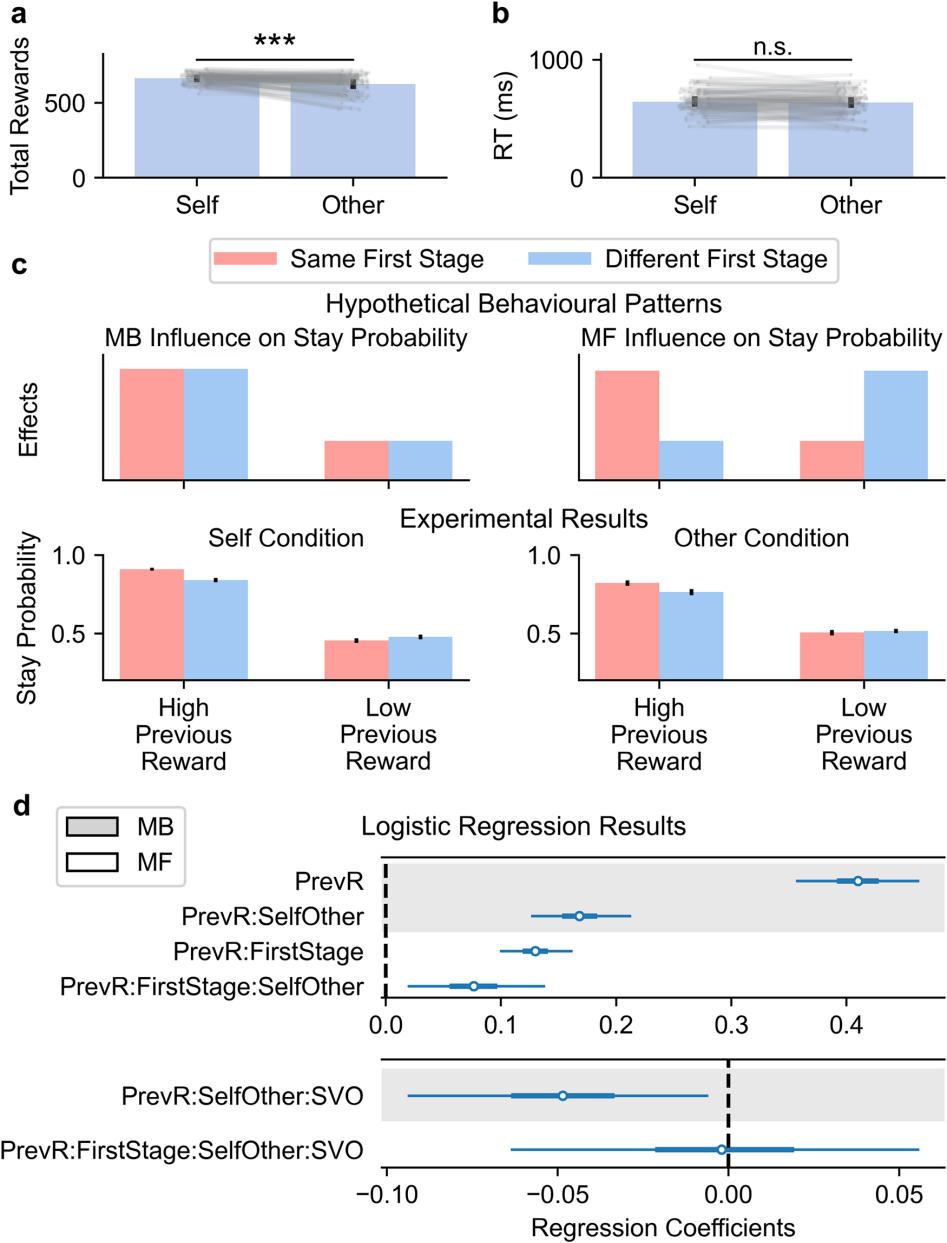
Model agnostic analysis of model-based and model-free systems. **a** Self-other differences in earned rewards. Grey lines and dots represent participants’ individual data points. The black error bars represent the standard error of the mean (SEM). The asterisk indicates statistical significance; ***: *p* < 0.001, n.s.: *p* > 0.05. **b** Self-other differences in the reaction time. **c** The upper panel is the hypothetical behavioural patterns associated with the model-based and model-free systems regardless of recipients, and the lower panel is the experimental results in Self and Other conditions. The stay probability, defined as the tendency to revisit the same planet, as a function of first-stage state similarity (red representing the same first-stage states, and blue representing different first-stage states) and previous outcome, was presented. The model-based behavioural signature was evidenced by the main effect of previous outcome (the upper left panel). In contrast, the model-free behavioural signature was indicated by the interaction between first-stage state similarity and previous outcome (the upper right panel). High and low previous rewards were determined based on whether previous rewards were larger than five or not. The black error bar represents the standard error of the mean (SEM). **d** The hierarchical Bayesian logistic regression results, with stay probabilities as the response variable. The posterior distributions of the regression coefficients were represented as bars. The thinner lines are 95% HDI, the thicker lines are interquartile range (IQR), and the empty circles are posterior means. The dashed lines point out the reference value (zero) of regression coefficients. Regression coefficients related to model-based systems were displayed on the grey background, whereas those related to model-free systems were presented on the white background. PrevR previous outcome, FirstStage first-stage state similarity, SelfOther Self/Other conditions, SVO individual variability in z-scored SVO.

To start with, we conducted paired sample *t*-tests on participants’ task performance to test whether they performed differently when deciding for themselves versus for another participant. We found that deciding for oneself was associated with significantly higher reward scores than deciding for others, *t*(91) = 6.68, *p* < 0.001, 95% CI [26.64, 49.16], *d* = 0.75 (Fig. 3a, total rewards for self: *M* ± *SD* = 662.05 ± 33.09; rewards for others: *M* ± *SD* = 624.15 ± 62.83. The time required to make decisions was comparable between the two recipients, *t*(91) = 0.95, *p* = 0.344, 95% CI [–7.44, 21.10], *d* = 0.07 (Fig. 3b, RTs for self: *M* ± *SD* = 645.90 ± 97.79 ms; RTs for others: *M* ± *SD* = 639.07 ± 103.26 ms).

Next, we used a hierarchical Bayesian logistic regression model to capture the behavioural signatures of model-based and model-free systems. We reported 95% Highest Density Interval (HDI) of the posterior distributions over regression coefficients. If the 95% HDI of a given regression coefficient did not include zero, this regression coefficient was considered significant. The logistic regression model defined the stay probability of repeating a visit to the same planet as the dependent variable, previous outcome, and similarity of first-stage states as the regressors. Specifically, the behavioural signature of model-based strategies could be characterised by the main effect of the previous outcome. A model-based learner would visit the same second-stage state if this state had yielded a satisfactory outcome on the previous trial, regardless of encountering which first-stage states. Because they could always use the cognitive map of the task structure to guide their decisions to the same second-stage state (the upper left panel of Fig. 3c). In contrast, the behavioural signature of model-free strategies could be characterised by the interaction term between first-stage state similarity and the previous outcome. Since model-free learners solely learnt the association between the previous first-stage state, their choice and the reward outcome, they would be less likely to revisit the same second-stage states if they encountered a different first-stage state, compared to when they encountered the same first-stage state (the upper right panel of Fig. 3c).

The logistic regression model (Fig. 3d) revealed a significant positive main effect of previous outcome on stay behaviours, *b*_*PreviousOutcome*_ = 0.41, 95% HDI [0.36, 0.46], indicating the behavioural signature of model-based strategies. The model also showed a positive interaction between first-stage state and previous outcome, *b*_*PreviousOutcome×FirstStage*_ = 0.13, 95% HDI [0.10, 0.16], suggesting the contribution of model-free strategies. As for the self-other differences, we found that deciding for oneself was linked to higher reliance on both systems than deciding for others (model-based influence on stay probabilities: *b*_*PreviousOutcome×SelfOther*_ = 0.17, 95% HDI [0.13, 0.21]; model-free influence on stay probabilities: *b*_*PreviousOutcome×*__*FirstStage×SelfOther*_ = 0.08, 95% HDI [0.02, 0.14]), suggesting that participants invested more cognitive effort for their own stake, compared to for another person’s interest. Other group-level regression coefficients were reported in the Supplementary Information.

To further test whether individual differences in SVO modulate self-other differences in the use of model-based and model-free strategies, we included z-scored SVO and all interaction terms among the previous outcome, first-stage state similarity, Self/Other conditions, and SVO as additional regressors into the hierarchical Bayesian logistic regression model (see Supplementary Table 2 for details of all group-level regression coefficients). The model pointed out that individual variability in SVO modulated self-other differences in the reliance on model-based strategies, *b*_*PreviousOutcome×SelfOther×SVO*_ = –0.05, 95% HDI [-0.09, -0.01] (the lower panel of Fig. 3d). More prosocial participants demonstrated less self-other differences in the use of model-based strategies than more proself participants. In contrast, SVO didn’t influence the self-other difference in model-free strategies, *b*_*PreviousOutcome×FirstStage×SelfOther×SVO*_ = –0.00, 95% HDI [-0.06, 0.06]. These results are consistent with our hypothesis that individual differences in SVO scores modulated self-other differences in the reliance on model-based strategies. Other group-level regression coefficients and their significance in modelling staying behaviours were reported in the Supplementary Information.

### Computational modelling results

To unfold the cognitive computations underlying the prosocial goal-directed behaviours, we formalised participants’ decisions as evidence accumulation processes using the RLDDM. Under this computational framework, the value difference between two available actions drove participants’ evidence gathering. Once the accumulated evidence hit one of the decision boundaries, the decision was made. The value difference was determined by several distinct cognitive mechanisms, including model-based, model-free state-action value estimates, choice stickiness and response stickiness. According to our parameter recovery analysis, all the parameters were well recovered (Fig. 2b, all correlation coefficients > 0.86) with overall low intercorrelations between these recovered parameter estimates (Supplementary Fig. 1a, the absolute values of correlation coefficients < 0.33). The high identifiability of the model parameters suggests that our model successfully captures both potential self-other differences and individual variability in these parameters. To further validate the absolute model fit, we performed a posterior predictive check by simulating 50 data sets (posterior predictive samples) using the estimated model parameters from the empirical data. The simulated data reproduced the following key behavioural patterns: (1) The simulated rewards earned by each agent replicated individual differences in the empirical rewards (Supplementary Fig. 2a). (2) The RT distributions generated by the RLDDM matched with the empirical RT patterns, i.e., increasing value differences between two options led to relative increases in choice probabilities for high-valued options and relative decreases in the corresponding RTs (Supplementary Fig. 2b). (3) After performing the same model-agnostic analysis on simulated samples, we found that the logistic regression results on posterior predictive samples (Supplementary Fig. 2c) greatly resembled empirically observed behavioural signatures (Fig. 3d).

**Fig. 4:**
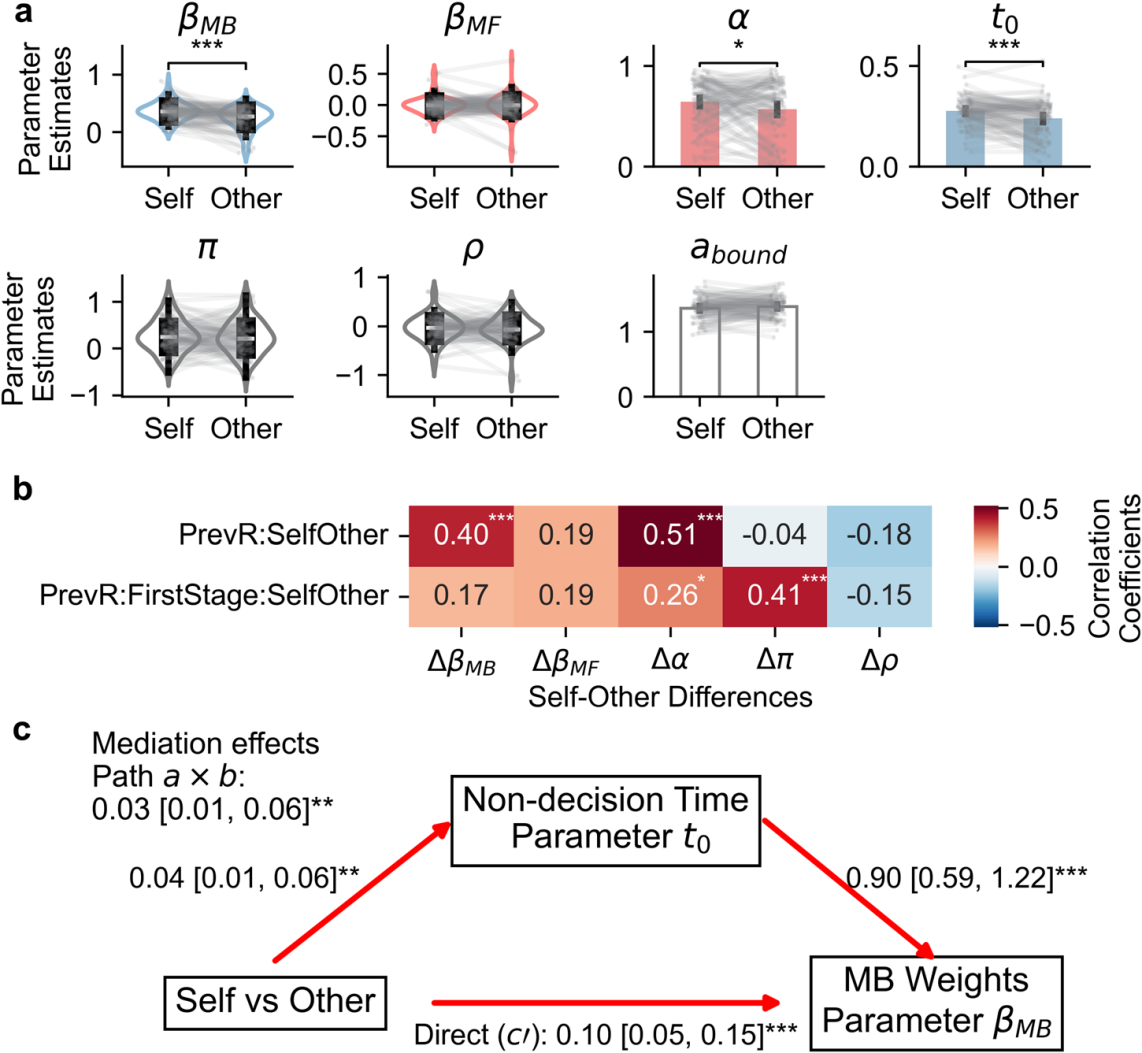
RLDDM results. **a** Self-other differences in the model parameters. Unbounded parameters were shown as violin plots, whereas bounded parameters were presented as bar plots. Grey lines and dots indicate participants’ individual data points. The violin plots demonstrate the median values (white lines), IQR (box-like structure), and whiskers (1.5 IQRs of the lower and upper quantiles). The error bars of bar plots represent the SEM. Significant self-other differences are highlighted by asterisks. ***: *p* < 0.001, **: *p* < 0.01, *: *p* < 0.05. **b** The correlations between self-other differences in model parameters (x-axis) and relevant behavioural signatures (y-axis). PrevR:SelfOther indicates the interaction effect of previous outcome and Self/Other conditions on stay probabilities, whereas PrevR:FirstStage:SelfOther represents the three-way interaction effect on stay probabilities. Significant coefficients were highlighted in white and accompanied by asterisks. **c** The mediation analysis result. We show the coefficients and 95% CI of each path. Significant path coefficients are marked with asterisks and presented as red arrows.

The comparison of computational parameters of the RLDDM between two recipients (Fig. 4a) revealed significant self-other differences in model-based weights, *t*(91) = 6.06, *p* < 0.001, 95% CI [0.09, 0.17], *d* = 0.71 (model-based weights for self: *M* ± *SD* = 0.37 ± 0.16; model-based weights for others: *M* ± *SD* = 0.24 ± 0.20). This result was consistent with the previous model-agnostic analysis. However, we observed no self-other differences in model-free weights, *t*(91) = 0.46, *p* = 0.645, 95% CI [-0.03, 0.05], *d* = 0.06. Instead, we observed significantly higher model-free learning rate when deciding for oneself than for others, *t*(91) = 2.28, *p* = 0.025, 95% CI [0.01, 0.14], *d* = 0.29 (model-free learning rate for self: *M* ± *SD* = 0.65 ± 0.22; model-free learning rate for others: *M* ± *SD* = 0.57 ± 0.28). We also found no self-other differences in choice and response stickiness (choice stickiness: *t*(91) = 0.48, *p* = 0.636, 95% CI [–0.07, 0.11], *d* = 0.06; response stickiness: *t*(91) = 0.27, *p* = 0.786, 95% CI [–0.06, 0.08], *d* = 0.03). Recall that the previous logistic regression model revealed significant self-other differences in the involvement of the model-free system. The current computational model further indicated that this discrepancy resulted from the decreased model-free learning rate, instead of the contribution of model-free value estimates. Furthermore, the lower prosocial model-free learning rate was not an artefact of heightened perseveration or motor repetition when deciding for others, as evidenced by comparable choice and response stickiness across two conditions. As for the remaining model parameters, deciding for self was associated with longer non-decision time, *t*(91) = 4.71, *p* < 0.001, 95% CI [0.02, 0.05], *d* = 0.47 (non-decision time for self: *M* ± *SD* = 0.28 ± 0.07 s; non-decision time for others: *M* ± *SD* = 0.24 ± 0.09 s). No significant self-other difference was found for the decision boundary, *t*(91) = –1.65, *p* = 0.102, 95% CI [–0.06, 0.01], *d* = 0.18.

Next, to test how parameter differences across two recipients were linked to self-other discrepancies in model-based and model-free behavioural signatures, we conducted correlation analyses (Fig. 4b). We found that prosocial model-based behavioural patterns (regression coefficients of PreviousOutcome:SelfOther) were positively correlated with self-other differences in model-based weights (*r*(92) = 0.40, *p* < 0.001, 95% CI [0.21, 0.56]) and model-free learning rates (*r*(92) = 0.51, *p* < 0.001, 95% CI [0.34, 0.65]). We found no correlation between self-other effects on model-based weights and model-free learning rates, *r*(92) = 0.00, *p* = 0.992, 95% CI [–0.20, 0.21], implying distinct cognitive computations underlying these effects despite similar behavioural signatures. In contrast, the prosocial model-free behavioural patterns (regression coefficients of PreviousOutcome:FirstStage:SelfOther) were positively associated with self-other differences in model-free learning rates (*r*(92) = 0.26, *p* = 0.013, 95% CI [0.06, 0.44]) and choice stickiness (*r*(92) = 0.41, *p* < 0.001, 95% CI [0.23, 0.57]). Similarly, we observed no significant correlations between self-other effects on model-free learning rates and choice stickiness, *r*(92) = –0.17, *p* = 0.102, 95% CI [–0.36, 0.03]. Together with the group-level effects shown in Fig. 4a, these results indicate that the identified self-other discrepancies in model-based weights and model-free learning rates predicted prosocial model-based and model-free influences on stay probabilities at both the group and participant levels. To further deepen our understanding of how these two model parameters give rise to prosocial model-based choices, we conducted a simulation study where self-other effects on either model-based weights, model-free learning rates, or both were selectively muted. After performing the logistic regression analysis on simulated data sets, we confirmed that prosocial model-based choices were driven by both model-based weights and model-free learning rates (see Supplementary Fig. 3), consistent with the correlation analyses.

Note that the higher non-decision time for oneself may explain the increased self-related model-based weight, as the longer non-decision time can reflect retrieving the mental model of the task transition structure and planning within it. Indeed, an exploratory mediation analysis (Fig. 4c) confirmed that self-other differences in non-decision time partially mediated the heightened self-related model-based weights compared to those associated with other-related decisions. To ensure that the mediation result was not confounded by individual variability in SVO scores, we repeated the mediation analysis while including SVO scores as a control variable. The mediation pathway remained significant (Supplementary Fig. 4). Further individual difference analysis indicated that those spending more non-decision time for others also demonstrated increased prosocial model-based weights (partial correlation after controlling SVO scores: *r*(92) = 0.38, *p* < 0.001, 95% CI [0.19, 0.54]).

### The relationship between SVO and model-based weights

**Fig. 5:**
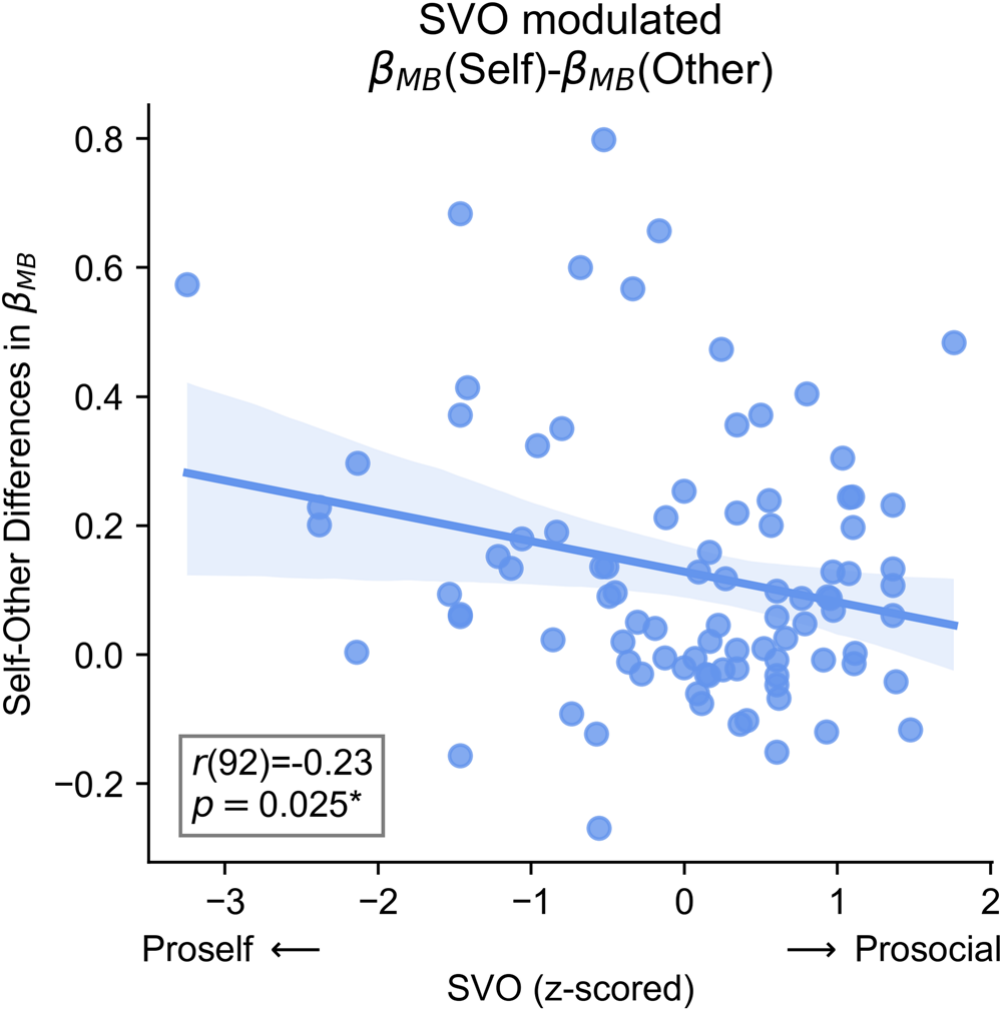
SVO modulated self-other differences in model-based weights. The scatter plot comparing SVO scores and self-other differences in model-based weights. Dots represent individual estimates. The solid line is the regression line. The shaded area represents the 95% CI. The two ends of prosocial and proself are shown on the x-axis.

We then performed correlation analysis to investigate the relationship between self-other differences in model-based weight and individual differences in SVO. The correlation analysis replicated the modulating role of SVO (Fig. 5), showing a significant negative correlation between self-other difference in model-based weight and SVO, *r*(92) = –0.23, *p* = 0.025, 95% CI [–0.42, –0.03]. This result revealed that more prosocial participants exhibited less self-other differences in model-based weight, suggesting that they were more willing to exert cognitive effort for others. To confirm the specificity of the link between self-other differences in model-based weight and SVO, we also performed post-hoc correlation analyses between SVO and self-other differences in all the remaining parameters, including model-free weight, model-free learning rate, non-decision time, decision boundary, choice stickiness, and response stickiness. No significant correlation coefficient was observed for self-other differences in the remaining parameters (the absolute values of correlations < 0.18, *p*s > 0.05).

## Discussion

To effectively benefit others in the ever-changing environment, people should not only learn how their actions may influence others, but also embed this association within a mental model of the environment. Our findings revealed both similarities and differences in the use of model-free and model-based strategies when deciding for others. For the model-free system, participants continued to rely on model-free values in their decisions (similar model-free weights across two recipients), but they updated those values more slowly for others than for themselves, evidenced by decreased model-free learning rate for others. This suggests that while habitual reinforcement processes remained engaged in prosocial contexts, the adaptation to recent outcomes became slower.

In contrast, model-based decision-making showed a more pronounced self-other discrepancy. Across both model-agnostic and computational analyses, participants were less likely to generalise outcomes across different states when making decisions for others, indicating reduced reliance on a forward-looking mental model. The RLDDM further clarified this difference: shorter non-decision time in the other condition partially accounted for the reduction in model-based weight, suggesting that people may be less willing to retrieve or apply a mental model on behalf of others. Finally, individual differences in social value orientation further shaped these effects, with more prosocial participants showing smaller self-other gaps in model-based decision-making than proself participants.

It is important to note that while the model-based weight in our computational model reflects the influence of a model-based learner on evidence accumulation speed, the observed self-other difference in this parameter does not inform differences in how mental models are learned. This interpretation is also consistent with how participants engaged with the task: First, participants had been extensively instructed on the task structure during the practice. Specifically, before entering the experiment, they had to choose the correct spaceship ten consecutive times when given the destination (i.e., the specific planet), ensuring their understanding of the task structure knowledge. Second, participants made decisions for two recipients based on the identical sets of stimuli, and the order of the two recipient conditions was counterbalanced. These settings suggest that the learnt mental model influenced value estimates equally across the two conditions. To sum up, the self-other effects on model-based weight captures changes in the extent to which model-based value estimates are integrated into the evidence accumulation process, rather than the distinct mental model learning processes.

Given that model-based decision-making is associated with an intrinsic subjective cost^9,49,50^, the reduced model-based decision-making for others can reflect the cost-benefit analysis weighing its cognitive cost against the potential rewards^9,12^. In this sense, participants may consider the cost of exerting model-based decision-making outweighing the subjective value of earning rewards for an anonymous future participant. Thus, they downregulated the use of model-based strategies for other-related choices. This explanation is aligned with previous studies suggesting people’s declined willingness to exert cognitive effort^17^ and conduct effortful information processing for others^29^. Similarly, the prosocial motivation of model-based decision-making is also consistent with motivational-intensity theory^51^, positing that effort investment is justified by the possibility of task success and its worth. Our findings suggest that people (at least the university students in our sample) are prosocial apathetic in terms of investing cognitive effort for others’ stake, generalising previous findings across effort modality^14,15^ and task contexts^16,17^.

The adoption of a process-modelling approach yielded considerable insights into the cognitive computations underlying prosocial model-based and model-free decision-making. While logistic regression models suggest a decrease in the model-free component for others, the RLDDM indicates that this self-other difference actually manifested as the reduced model-free learning rate, rather than by a diminished contribution of model-free value estimates, increased perseveration, or greater motor repetition, consistent with previous findings^16,52^. Similarly, the logistic regression models also suggest a reduction in the model-based component for others. Such behavioural patterns were associated with a diminished contribution of model-based value estimates and slower model-free learning speed, as revealed by the RLDDM. Critically, the unique value of formalising decisions as evidence accumulation processes in this study lies in its ability to isolate the non-decision time. This decomposition allowed us to directly link the non-decisional process to prosocial model-based decision-making through a mediation model. This mediation model can reflect the process of retrieving internal memory representation, i.e., the mental model of task transition structure, to guide model-based value estimates^53^. Corroborating this explanation, individual difference analysis further revealed that spending more time on others was associated with increased prosocial model-based decision-making. These findings contribute to the recent debate about whether intuitive or reflective decision-making promotes prosocial behaviour^54,55^. Specifically, the longer deliberation to consult the mental model and perform prosocial model-based decision-making supports that reflective decision modes facilitate the prosociality^29,54,56^. Collectively, our results highlight the power of computational modelling in uncovering the computations underlying social cognition^57,58^.

A plethora of research has shown that SVO is associated with behaviours in various social contexts, including pro-environmental behaviour^59,60^, political preferences^61,62^, donation^63^, data sharing^64^, risky decision-making for others^41,65^, and neural responses related to outcome evaluation for oneself and others^42^. We extend this line of research by showing the negative correlation between SVO and self-other differences in model-based decision-making. Considering that cognitive resources for decision-making are limited^66,67^, this link may reflect proselfs’ and prosocials’ distinct cognitive resource allocation strategies. Two potential cognitive mechanisms may explain our findings. First, the negative correlation may result from distinct inequality aversion for prosocials and proselfs. According to the integrative model of SVO^68,69^, prosocials aim to achieve an equal resource distribution and maximise joint outcomes, whereas proselfs only concern their own interest and care less about equality. Both behavioural and neural evidence confirmed this prediction, with prosocials primarily concerning the fairness in the allocation of financial resources^70–72^. In line with this notion, we observed that the linear relationship between SVO and self-other differences in model-based weights demonstrated a close to zero discrepancy when SVO approaching the prosocial end (see the regression line in Fig. 5). Alternatively, selfish individuals may rely more on cognitive control to make prosocial decisions^73^, whereas prosocial individuals may be more willing to behave prosocially, without the need of cognitive control^74,75^. In this sense, proself individuals tend to assign more subjective cost to prosocial model-based decision-making, leading to decreased engagement in model-based decision-making for others. Taken together, our results suggest the correspondence between financial resource allocation and cognitive resource allocation between oneself and others.

The present study focused on whether and how model-based and model-free decision-making are engaged when people make decisions for themselves versus for others, and on how individual differences in SVO shape these self–other differences. It is important to acknowledge, however, that many additional factors—such as social distance and the recipient’s physical presence—may also influence prosocial model-based decision-making. For example, Navidi et al.^19^ reported no self-other differences when the recipient was physically present in the same room. In contrast, our participants made decisions for an anonymous future participant, a context that likely induces relatively lower prosocial motivation. This setting reduces situational influences on prosocial behaviour, which may allow stable individual differences such as SVO to exert a clearer influence on how people make decisions for others. Nevertheless, it remains important for future work to test how SVO modulates behaviour across contexts with varying levels of social distance. As noted in the Introduction, prior studies have reported mixed findings regarding whether model-based decision-making shifts in prosocial contexts, and recipient presence has been proposed as a potential moderator. Because we did not manipulate social distance directly, our findings cannot fully adjudicate this mixed literature. Instead, they complement existing work by providing a new lens—via the RLDDM—to reveal the latent cognitive computations underlying prosocial model-based decision-making and how these processes relate to SVO.

This study also has several limitations. First, while our study demonstrated the self-other effects on the use of model-based strategies, it didn’t explore how the mental model learning processes might differ when people make decisions for themselves versus others. Previous studies^76–78^ have shown that structural learning is cognitively demanding, due to the effort required in selecting candidate abstract rules through hypothesis testing. Given that both our study and previous literature^14–17^ have demonstrated people’s unwillingness to exert effort for others, it is plausible that people may learn task structure more slowly when learning on behalf of others. Additionally, those who spend more time to learn for others might perform better in other-related learning, as we found similar results that longer non-decision times for others were associated with increased use of model-based strategies in other-related decisions. Future studies could adapt a variant of the two-step task and use behavioural representational similarity analyses^79^ to directly examine the quality of mental model learning for oneself vs others. Second, while our models are sufficient to capture the key behavioural signature of model-based decision-making^79^, i.e., forward simulation of potential destinations, the dual-system distinction of model-based and model-free systems may not capture the full spectrum of strategies determining participants’ behaviours^80,81^. This can be especially the case for prosocial decisions, considering the high decision noise when making decisions on behalf of others^29,30^. Future studies could incorporate more strategies, such as higher-order perseveration^82^, and variants of DDM, like Levy flight model^83^, into the computational models to uncover the underlying decision-making processes. Third, the mechanisms underlying the relationship between SVO and prosocial model-based decision-making are speculative. Although we have discussed two potential cognitive mechanisms underlying this link, whether these two factors drive the link together or separately remains an open question. Thus, further investigation is required to disentangle these mechanisms. Finally, considering the length of the two-step task and our aim of probing prosocial motivation, it was not feasible to include more recipients, such as close others and a charity. While recent studies have shown that social decision-making varies as a function of closeness, such as distinct recipients^17,84–87^, whether model-based decision-making will also demonstrate this pattern is unclear. Future studies can directly include these recipients and make use of a between-subject design to address this question. However, as deciding for a completely anonymous person is common in this digital society, such as sharing experiences and answering other people’s questions on social media, our findings carry essential implications for our understanding of how people may allocate cognitive resources to plan for others.

In conclusion, using the RLDDM, we demonstrated that deciding for others significantly decreased the involvement of model-based strategies and slowed down the model-free learning, suggesting the diminished investment of cognitive effort for others. In particular, the decreased use of model-based strategies was partially mediated by the self-other difference in non-decision time, revealing relevant memory-based decision processes of prosocial model-based decision-making. Finally, we observed that SVO modulated self-other differences in model-based decision-making, with prosocials showing less self-other discrepancies than proselfs. These results unravel the cognitive computations underlying prosocial model-based decision-making, pinpointing the potential shared mechanisms between financial resource allocation and cognitive resource allocation.

## Methods

### Participants

We recruited 107 healthy younger adults (range: 18–39 years old) from the community around the City University of Hong Kong. We excluded 15 participants according to the following criterion: (1) 3 participants were excluded because of missing SVO data; (2) 4 participants were excluded due to more than 20% invalid trials, including both missing trials and extremely fast trials (response times < 200 ms); (3) 3 participants were excluded because they didn’t believe that they made decisions for another participant, evidenced by their answers to post-experiment open questions; (4) 5 participants were excluded because they responded randomly throughout the experiment. Specifically, we determined this using a two-step procedure. First, we excluded 2 participants who explicitly reported that they simply employed a random choice strategy. Next, we further filtered out 3 participants by simulating 1000 random agents selecting first-stage responses randomly^79^. This simulation generated a null distribution of negative log-likelihoods corresponding to random action selection for each block. The cut-off threshold was determined by the 5th percentile of this distribution (Block 1: 38.770, Block 2: 38.532, Block 3: 38.461, Block 4: 39.317). Participants whose negative log-likelihood scores exceeded these thresholds in both self-related blocks and other-related blocks were excluded. These criteria led to a total of 92 effective samples (58 female, 32 male, 2 unindicated, age: *M* = 24.52, *SD* = 3.93, ranging from 18 to 39). This sample size was determined using the R (version 4.5.1) package pwr^88^ (version 1.3.0), which would achieve approximately 90% power to detect a medium-size effect (*d* = 0.3) in potential self-other differences with a significance level of 0.05 (one-sided). All participants gave written informed consent and reported no history of psychiatric and neurological disorders. The study procedures were approved by the Human Subjects Ethics Sub-committee of the City University of Hong Kong and were conducted in accordance with the Declaration of Helsinki. Participants were compensated for their participation and received an additional monetary bonus according to their task performance.

### Materials and procedure

After providing informed consent, participants were instructed to complete demographic questionnaires. Then, they performed a modified two-step task^9^, aiming at probing how self-other differences impact mental effort investment (Fig. 1a). We manipulated the self and other conditions by explicitly instructing participants that they would decide for both themselves and an anonymous future participant in this task^89^. In self-related blocks, outcomes of their decisions would lead to their own gains. In contrast, in other-related blocks, the scores they obtained would be attributed to another participant. The obtained scores in the task would be transformed into extra bonus with a ratio of 20:1, i.e., each 20 scores equal 1 Hong Kong dollar (HKD). Participants would know nothing about the person for whom they made decisions, except for that this person would also take part in this study in the future. And they were informed that the recipient would know nothing about them. After the completion of this task, participants were asked to finish the SVO Slider Measure^90^, as well as open questions regarding how they felt about the recipient and the task and the strategy they used.

The SVO Slider Measure is an efficient and simple measure of SVO, with great convergent validity^90^, high test-retest reliability^91,92^, and a considerable degree of stability over a long period^93^. It contains six primary items and nine secondary items. We used the six primary items to estimate individual differences in SVO scores. For each item, participants were asked to determine a preferred distribution of hypothetical money between themselves and an anonymous other. The joint monetary outcomes ranged between 100 and 170 HKD. Participants’ allocation choices were then mapped onto a two-dimensional space with their own money and another person’s money as two axes. Individual SVO scores were calculated as the inverse tangent of the ratio between mean self-related payoffs minus 50 and mean other-related payoffs minus 5^90^. This procedure yielded a continuous measure of SVO scores ranging between −16.26° and 61.39°, with larger values indicating more prosocial orientations.

The two-step task comprised two stages per trial: the first stage involving spaceships and the second stage involving aliens (Fig. 1a). Each trial randomly started from one of the two possible first stages, each characterised by different pairs of spaceships. Participants were required to choose one of the two spaceships within a time limit of 1500 ms by pressing “F” (the left spaceship) and “J” (the right spaceship). Within each pair of spaceships, the presentation positions (left or right) were randomised. Following their selection, they transitioned to the second stage based on the arrows depicted in Fig. 1b, where a red or purple alien appeared on the screen. This transition was deterministic, meaning that choosing a particular spaceship consistently leads to the same alien. After arriving at one of the two planets and meeting the alien, to reveal and receive rewards, participants were asked to press the spacebar within a response period of 1500 ms. The alien on each planet delivered “space treasures” only if the keypress was successfully registered. Otherwise, the aliens would provide zero outcome. These “space treasures” would then be transformed into scalar rewards, contributing to the total reward scores (Fig. 1a). Reward outcomes were initialised randomly as low values (0–4) for one planet and high values (5–9) for the other. To encourage learning throughout the task, the outcomes of each planet randomly drifted according to two independent Gaussian random walks (*σ* = 2), reflecting at boundaries (0 and 9). After pressing the button at each stage, the chosen option would be highlighted with a white frame for the rest of the response period. This way ensures that participants cannot increase their reward rate per unit time by responding more quickly. Trials with missing first-stage responses would lead to zero outcome and were discarded from the analysis. As for the self and other conditions, the recipient of each block was cued to participants at the beginning of each block (Fig. 1a). The recipient of each trial was also presented above the stimuli throughout each trial.

Prior to the formal two-step task, participants were extensively instructed on the task transition structure, the outcome distribution and the manipulation of the recipients. They were shown the transition structure of spaceships and instructed to arrive at a given planet by choosing the correct spaceship. Only those who consecutively chose 10 correct spaceships could continue the task, ensuring that they clearly knew the task transition structure. Furthermore, they had to correctly report who would get the rewards when the recipient appeared above the stimuli. Next, they finished 25 practice trials. The only difference between the practice trials and the formal task was that the obtained scores would not be transformed into bonus money. The formal two-step task contained 4 blocks (2 self-related blocks and 2 other-related blocks) with each block consisting of 60 trials, resulting in a total of 240 trials. Between every two blocks, participants would have a self-paced break (maximum 2 min).

Following the previous study^12,94^, to decrease the between-participant variability in task performance, two pre-determined trial sequences were created, including the sequences of first-stage states, second-stage outcomes and self-other conditions. The first-stage states and two second-stage payoff trajectories were identical for these two trial sequences, whereas the self-related and other-related blocks were counterbalanced. Each participant would randomly perform one of the two trial sequences.

Notably, this task effectively distinguishes between model-based and model-free strategies (Fig. 1c). Individuals fully employing a model-based strategy choose the first-stage spaceship based on the previous reward outcome, irrespective of which specific first stage they are in. Because model-based strategies allow them to predict the outcome according to the mental representation of task structure. On the other hand, people following a pure model-free strategy cannot generalise their experience from one pair of spaceships to another pair. Their learning is confined to specific action-reward associations.

To illustrate further, consider a player who experienced the trial sequence depicted in Fig. 1c. In a given trial *T-2*, the player selected the orange spaceship, transitioned to the purple planet, and obtained 5 scores. In the subsequent trial *T-1* starting with another pair of spaceships, the player selected the green spaceship, transitioned to the red planet and obtained a better reward of 8 scores. In the next trial *T*, if the player employed a pure model-based strategy, the player would choose the cyan spaceship to reach the red planet based on their mental representation of the state transition knowledge. On the contrary, a player fully following a model-free strategy would not exhibit this tendency. Instead, they would choose the orange spaceship according to the reward history (trial *T-2*). This distinction allows us to differentiate between model-based and model-free strategies.

### Behavioural analysis

For the self-other differences in the total obtained rewards and reaction times (RTs), we conducted paired sample *t*-tests. For the model-agnostic analysis of model-based and model-free strategies, hierarchical Bayesian logistic regression models were employed. As described in the previous section, these two kinds of strategies demonstrate different behavioural signatures, which were well captured by hierarchical Bayesian logistic regression models. Specifically, the dependent variable was the stay probability of repeating a visit to the same second-stage state (the same planet/alien), coded as 1 if the current second-stage state was the same as the previous trial, 0 otherwise. The reward outcome of the previous trial and similarity in first-stage states, as well as their interaction term, were defined as the regressors. Similarity in first-stage states was coded as 1 if participants encountered the same pair of spaceships on the current and the previous trial, coded as 0 otherwise. The behavioural signature of model-based and model-free strategies could be characterised by the main effect of previous outcome and the interaction term between first-stage states and previous outcome, respectively. We visualised these two effects in Fig. 3c, using the following stay probabilities: high stay probability = 0.90 and low stay probability = 0.45. These values are hypothetical and are used solely for illustration purposes.

To test the self-other differences, the hierarchical Bayesian logistic regression model was implemented using Bambi (BAyesian Model Building Interface)^95^ package (version 0.15.0) in Python (version 3.11.11), with the following syntax: Stay ~ PreviousOutcome * FirstStage * SelfOther + (PreviousOutcome * FirstStage * SelfOther | ID), where self and other conditions were effect coded (self: 0.5, other: -0.5). The modulation role of SVO scores was examined by including individuals’ z-scored SVO scores into the model: Stay ~ PreviousOutcome * FirstStage * SelfOther * SVO + (PreviousOutcome * FirstStage * SelfOther | ID). Weakly informative default priors were used. We ran 4 independent Markov chains with 2500 iterations each to estimate the posterior using the No U-Turn Sampler (NUTS)^96^. The first 500 samples were discarded as the warm-up, resulting in 8000 posterior samples.

### Computational modelling analysis: Reinforcement Learning Drift-Diffusion Model (RLDDM)

We employed a well-established reinforcement learning drift-diffusion model (RLDDM)^20,21,28^ to describe participants’ behaviours when they made goal-directed decisions for themselves and for others. Similar to the model-agnostic analysis, modelling the first-stage responses allows the quantification of the extent to which participants followed model-based and model-free strategies. This model consists of two components, as shown in Fig. 2a: a model-free learner and a model-based learner^12,94^. The state-action values *Q*(*s*, *a*) of each state-action pair, defined as how good choosing the action *a* is when in the state *s*, were the weighted sum of these two components. For each trial *t*, there are two possible first-stage states (*s*_*1,t*_) with two available actions (*a*_*1,t*_) and two second-stage states (*s*_*2,t*_) with only one possible action (*a*_*2,t*_).

#### Model-free learner

The model-free learner updates the state-action values based on the temporal difference learning. The reward prediction error signals, the discrepancy between the reward expectation and actual reward outcome, were used to update the first-stage and second-stage state-action values. For each stage, the reward prediction error signals (*δ*_*1,t*_ and *δ*_*2,t*_) were calculated based on the following equations:

$${\delta }_{1,t}={Q}_{{MF}}\left({s}_{2,t},{a}_{2,t}\right)-{Q}_{{MF}}\left({s}_{1,t},{a}_{1,t}\right)$$

$${\delta }_{2,t}={r}_{t}-{Q}_{{MF}}\left({s}_{2,t},{a}_{2,t}\right)$$

Then the model-free learner incorporates these reward prediction errors to update the corresponding state-action value estimates:

$${Q}_{{MF}}\left({s}_{1,t},{a}_{1,t}\right)={Q}_{{MF}}\left({s}_{1,t},{a}_{1,t}\right)+\alpha {\delta }_{1,t}+\alpha \lambda {\delta }_{2,t}$$

$${Q}_{{MF}}\left({s}_{2,t},{a}_{2,t}\right)={Q}_{{MF}}\left({s}_{2,t},{a}_{2,t}\right)+\alpha {\delta }_{2,t}$$

The parameter *α* represents the learning rate, controlling the extent to which reward prediction errors are incorporated into the state-action value updates. The parameter *λ* denotes the eligibility trace decay, determining the influence of second-stage reward prediction errors on first-stage state-action value estimates. Both parameters fall within the range between 0 and 1. As the eligibility trace decay parameter showed relatively poor parameter recovery performance^28^, we set *λ* = 1.

*Model-based learner*. In contrast, the model-based learner estimates the first-stage state-action values by making use of the task transition structure *T*(*s*_*2*_ | *s*_*1*_, *a*_*1*_), which represents how first-stage states (*s*_*1*_) and second-stage states (*s*_*2*_) are connected according to the first-stage actions (*a*_*1*_). The trial subscript is not included here because the transition structure in our task is stable. The state-action values were then calculated by taking this transition structure into consideration:

$${Q}_{{MB}}\left({s}_{1,t},{a}_{1,t}\right)={\sum }_{{s}_{2}}{T({s}_{2}|{s}_{1},{a}_{1})\,Q}_{{MB}}\left({s}_{2},{a}_{2}\right)$$

$${Q}_{{MB}}\left({s}_{2,t},{a}_{2,t}\right)={Q}_{{MF}}\left({s}_{2,t},{a}_{2,t}\right)$$

Here, the second-stage model-based value estimates are the same as the model-free learner, because they both calculate the immediate rewards.

We assumed that the second-stage model-free state-action values were initialised as 4.5 (the average of minimum and maximum outcomes). *T*(*s*_*2*_ | *s*_*1*_, *a*_*1*_) = 1 if choosing *a*_*1*_ in *s*_*1*_ would lead to *s*_*2*_, otherwise *T*(*s*_*2*_ | *s*_*1*_, *a*_*1*_) = 0, corresponding to the task transition structure.

*Choice rule*. First-stage choices were formalised as evidence accumulation processes^97^. Participants gradually gathered noisy evidence about two alternative choices until the accumulated evidence hit one of the decision boundaries. The evidence accumulation process is defined as the following equation:

$${dx}=v{dt}+\sigma d\xi {with\; \xi } \sim {Normal}(0,1)$$

where *dx* represents the accumulated evidence at each time point *dt*, the drift rate parameter *v* denotes the rate of information uptake, and the noise term *ξ* denotes the stochastic noise (*σ* = 1) during evidence accumulation.

The evidence accumulation process starts from the initial point *z*, and terminates when the gathered evidence reaches 0 or the decision boundary parameter *a*. As we assumed no initial bias, we set *z* = 0.5*a. The drift rate parameter *v* is determined by the value difference between the two actions in the first-stage states, where both model-based and model-free value estimates exert their influences. The drift rate of choosing action *a*_*1*_ in the first-stage state *s*_*1,t*_ can be defined as the following:

$${v}_{{a}_{1,t}={a}_{1}|{s}_{1,t}}={\beta }_{{MB}}{Q}_{{MB}}\left({s}_{1,t},{a}_{1}\right)+{\beta }_{{MF}}{Q}_{{MF}}\left({s}_{1,t},{a}_{1}\right)+\pi \cdot {ch}\left({a}_{1}\right)+\rho \cdot {resp}\left({a}_{1}\right)$$

In this equation, *β*_*MB*_ is the model-based weights, *β*_*MF*_ is the model-free weights. These two unbounded parameters control the degree to which each strategy influences the evidence accumulation speed. Notably, while previous studies using a single weight parameter to describe the trade-off between two strategies^6,9^, the parameterisation we used here is algebraically equivalent to the conventional setting^94,98^. We considered unbounded model-based and model-free weight parameters for two reasons: (1) unbounded weight parameters facilitate the model fitting of the RLDDM; (2) this parameterisation can better account for the potential high decision noise when deciding for others.

The choice stickiness parameter *π* and the response stickiness parameter *ρ* describe the repeating or switching of the previous choice of spaceship (*π*) or previous key press (*ρ*), respectively. Positive parameter values indicate the tendency to repeat previous selections, whereas negative values indicate the tendency to switch to another choice or key. The indicator variable *ch*(*a*_*1*_) = 1 if the action *a*_*1*_ corresponds to the same spaceship selected in the previous trial, otherwise *ch*(*a*_*1*_) = 0. Similarly, *resp*(*a*_*1*_) = 1 if the action *a*_*1*_ corresponds to the same key pressed in the previous trial, otherwise *resp*(*a*_*1*_) = 0.

Under this framework, choices and RTs are jointly modelled. Choices reflect the accumulating evidence toward a certain decision boundary, whereas RTs indicate the time required to hit the decision boundary plus a constant non-decision time *t*_*0*_ (the time unrelated to decision processes, such as the time taken to encode the stimuli and execute motor responses).

To capture the potential self-other differences in the model parameters, we allowed all parameters to vary between two conditions, resulting in a total of 14 parameters with 7 parameters for each condition: model-based weight *β*_*MB*_, model-free weight *β*_*MF*_, model-free learning rate *α*, choice stickiness parameter *π*, response stickiness parameter *ρ*, decision boundary *a*, and non-decision time *t*_*0*_. Notably, in the other condition, we set *β*_*MB(Other)*_ = *β*_*MB(Self)*_ + *β*_*MB(Other-Self)*_ for both model-based weights and model-free weights, where self-other differences were explicitly set as the parameters to be estimated. Potential self-other differences in these parameters were tested using paired sample *t*-tests.

Model parameters were fit using Maximum a Posteriori (MAP) estimation. For model-based weight *β*_*MB*_, model-free weight *β*_*MF*_, choice stickiness parameter *π*, and response stickiness parameter *ρ*, their priors were standard normal distributions. For self-other differences in model-based and model-free weights, we used a normal prior Normal (0, 0.5). For the learning rate parameter *α*, beta distributions Beta (2, 2) were used. For the decision boundary *a*, we used gamma distribution Gamma (1.5, 0.75). For the non-decision time *t*_*0*_, we used gamma distribution Gamma (0.8, 0.2). Genetic algorithms were used to optimise the posterior, implemented using the Pygad^99^ package (version 3.3.1) in Python. For the genetic algorithms, the hyperparameters were defined as follows: The number of generations was set to 100; The number of solutions to be selected as parents in the mating pool was defined as 100; The number of solutions within the population was set to 200; The number of genes was the same as the number of parameters (i.e., 14); The search space of *β*_*MB*_, *β*_*MF*_, *π*, and *ρ* was set to [-3, 3], the search space of self-other differences in *β*_*MB*_ and *β*_*MF*_ was set to [-1, 1], the search space of *α* was set to [0.00001, 0.99999], the search space of decision boundary was set to [0.3, 3], the search space of *t*_*0*_ was set to [0.001, 1]. For other hyperparameters related to genetic algorithms, the defaults were used. To avoid local optima, we initialised the optimisation procedure with 10 different initial values and selected the run with the best posterior estimate.

To assess whether our model can reliably capture the self-other differences and individual differences in these parameters, we performed parameter recovery analysis (Fig. 2b). We simulated behaviours of 100 agents whose parameters were randomly drawn from the following uniform distributions: U(-0.5, 1) for *β*_*MB*_ and *β*_*MF*_, U(−1, 0.5) for self-other differences (Other-Self) in *β*_*MB*_ and *β*_*MF*_, U(0, 1) for *α*, U(−1.2, 1.2) for *π*, and *ρ*, U(0.9, 2) for decision boundary, and U(0.02, 0.55) for *t*_*0*_. The same uniform distributions were used for these parameters across two conditions, except *β*_*MB*_ and *β*_*MF*_. Importantly, these ranges of uniform distributions covered the parameter values estimated from the empirical data. We then attempted to recover the true parameters by fitting the simulated behaviours with our computational model. The Spearman correlation coefficients between the true parameters and the recovered parameters, as well as the intercorrelations among the recovered parameters, were reported.

A posterior predictive check was conducted by simulating 50 posterior predictive samples using the fitted parameter values. Each simulated data set consisted of 92 agents, corresponding to the actual number of participants. We calculated the correlation between the average simulated rewards and empirical rewards obtained by each agent. The RT distributions of the posterior predictive samples were also plotted as a function of recipients and value differences. Additionally, we employed the same logistic regression models reported in the model-agnostic analysis (Fig. 3d) to analyse these simulated data sets. To reduce the computational burden (estimating posterior distributions of regression coefficients for each simulated data set), the logistic regression models were estimated using a variational Bayes approach, as implemented in Bambi. The Automatic Differentiation Variational Inference (ADVI) with 15000 iterations was used to fit models. Per data set, posterior means of regression coefficients were recorded. We presented the summary statistics of posterior means of regression coefficients from 50 simulated data sets.

To link the prosocial model-based and model-free behavioural signatures with self-other differences in fitted parameters, we conducted correlation analyses. We calculated correlation coefficients between the participant-level regression coefficients on stay probabilities and self-other effects on model parameters. The participant-level effects were derived from the following hierarchical Bayesian logistic regression model: Stay ~ PreviousOutcome * FirstStage * SelfOther + (PreviousOutcome * FirstStage * SelfOther | ID). We obtained each participant’s coefficient estimates by calculating the posterior expectation. We selected the following participant-level effects due to their relevance to prosocial model-based and model-free behavioural patterns: PreviousOutcome:SelfOther (model-based component), and PreviousOutcome:FirstStage:SelfOther (model-free component). As for the model parameters of interest, we chose those related to the drift rate of the DDM (*β*_*MB*_, *β*_*MF*_, *α*, *π*, and *ρ*) because they influenced the evidence accumulation process and determined choices.

To further deepen our understanding of how these two model parameters give rise to prosocial model-based choices, we performed a simulation study using the parameters estimated from the empirical data. The simulation study was conducted in a similar way to the posterior predictive check. The only difference was the parameter set used to generate 50 simulated data sets. We selectively muted self-other effects on either model-based weights or model-free learning rates, or both, by setting these parameters to identical values across Self/Other conditions. The procedure resulted in three parameter sets: (1) only self-other differences in model-based weights were muted; (2) only self-other differences in model-free learning rates were muted; (3) self-other differences in both parameters were muted. Then the following regression model was employed to analyse these simulated data sets (a total of 150 data sets, 50 data sets for each parameter set): Stay ~ PreviousOutcome * FirstStage * SelfOther + (PreviousOutcome * FirstStage * SelfOther | ID). To reduce computational cost, regression coefficients were estimated using a variational Bayes approach, using ADVI with 15000 iterations. Per parameter set, we presented the summary statistics of posterior means of regression coefficients. These simulation results were compared to a baseline condition (posterior predictive check reported in Supplementary Fig. 2c), where self-other effects were preserved.

### Mediation analysis

To further explain potential self-other differences in model-based decision-making, we conducted exploratory mediation analysis to test whether self-other differences in non-decision time, the time related to non-decisional processes, such as visual encoding, memory retrieval and motor execution, can explain self-other differences in model-based decision-making. Considering that the retrieval of the mental model is a prerequisite of model-based decision-making^100^, the use of model-based strategies may introduce additional non-decision time. In this mediation model, the path from self vs other to non-decision time and the path from the non-decision time to model-based weights were simultaneously estimated. The product of the two coefficients is the indirect effect. The statistical significance of all coefficients was tested using bootstrapping of 10,000 iterations. Additionally, we repeated the same mediation analysis. But this time, the SVO scores were controlled as the covariate. All the mediation models were implemented using the Pingouin^101^ package (version 0.5.5) in Python.

## Supplementary information

Supplementary Information

## Data Availability

The data used in the study are available from the corresponding author upon reasonable request.

## Code availability

The code used in the study is available from the corresponding author upon reasonable request.
