# Supplementary material for: Deciding for others diminishes model-based decision-making but depends on individual prosociality
Source: NPJ Sci Learn. 2026 Jan 10;11:11. doi: 10.1038/s41539-025-00397-0 (PMC12881483; doi:10.1038/s41539-025-00397-0)
Supplement: Supplementary file 1 — Supplementary Information [file 41539_2025_397_MOESM1_ESM.pdf]

Deciding for Others Diminishes Model-Based Decision-Making but Depends on Individual Prosociality

Supplementary Information

Hierarchical Bayesian logistic regression results on stay probabilities

The logistic regression model revealed a significant positive intercept, b_0 = -1.42, 95% HDI [-1.68, -1.15], indicating that participants tended to switch to another planet when receiving zero reward. The model also showed a negative slope for the first-stage similarity, $b_{FirstStage}$ = -0.31, 95% HDI [-0.47, -0.16], suggesting the tendency to switch was more pronounced when the current first-stage state was the same as the previous trial. As for the self-other differences, we found that deciding for oneself was linked to a stronger tendency to switch, $b_{SelfOther}$ = -0.75, 95% HDI [-0.96, -0.53]. No self-other difference was found for the interaction term between the first-stage similarity and Self/Other conditions, $b_{FirstStageState \times SelfOther}$ = -0.30, 95% HDI [-0.63, 0.03].

The logistic regression model including SVO-related regressors replicated the same pattern as the model without SVO-related regressors, see Supplementary Table 2 for further details. As for SVO-related regressors, we observed a negative slope for SVO, b_{SVO} = -0.36, 95% HDI [-0.61, -0.11], suggesting that prosocials were more likely to switch to another planet when receiving zero reward. We also found a positive interaction effect between previous outcome and SVO, $b_{PreviousOutcome \times SVO}$ = 0.07, 95% HDI [0.01, 0.12], indicating that prosocials exhibited stronger behavioural signatures of model-based systems. No significant effect was found for other SVO-related regressors.

Supplementary Table 1. Regression coefficients from hierarchical Bayesian logistic regression analysis, indicating the effect of previous outcome, first-stage state similarity, and Self/Other condition, on stay probabilities.

Regression Coefficient	Mean	SD	HDI 2.5%	HDI 97.5%
(Intercept)	-1.426	0.133	-1.676	-1.153
SelfOther	-0.745	0.110	-0.960	-0.530
FirstStage	-0.313	0.081	-0.472	-0.159
FirstStage:SelfOther	-0.304	0.168	-0.627	0.029
PreviousOutcome:FirstStage	0.130	0.016	0.099	0.162
PreviousOutcome:FirstStage:SelfOther	0.076	0.031	0.019	0.138
PreviousOutcome	0.410	0.027	0.356	0.463
PreviousOutcome:SelfOther	0.168	0.022	0.126	0.213

Supplementary Table 2. Regression coefficients from hierarchical Bayesian logistic regression analysis, indicating the effect of previous outcome, first-stage state similarity, Self/Other condition, and SVO scores, on stay probabilities.

Regression Coefficient	Mean	SD	HDI 2.5%	HDI 97.5%
(Intercept)	-1.427	0.131	-1.680	-1.173
SVO	-0.356	0.128	-0.605	-0.107
SelfOther	-0.729	0.112	-0.940	-0.506
SelfOther:SVO	0.193	0.110	-0.018	0.415
FirstStage	-0.312	0.082	-0.475	-0.156
FirstStage:SVO	0.044	0.081	-0.112	0.203
FirstStage:SelfOther	-0.296	0.170	-0.642	0.022
FirstStage:SelfOther:SVO	0.092	0.165	-0.239	0.404
PreviousOutcome:FirstStage	0.130	0.017	0.098	0.162
PreviousOutcome:FirstStage:SVO	0.012	0.016	-0.018	0.043
PreviousOutcome:FirstStage:SelfOther	0.074	0.032	0.011	0.135
PreviousOutcome:FirstStage:SelfOther:SVO	-0.002	0.030	-0.064	0.056
PreviousOutcome	0.410	0.027	0.358	0.463
PreviousOutcome:SVO	0.066	0.026	0.014	0.118
PreviousOutcome:SelfOther	0.165	0.023	0.123	0.212
PreviousOutcome:SelfOther:SVO	-0.049	0.022	-0.094	-0.006

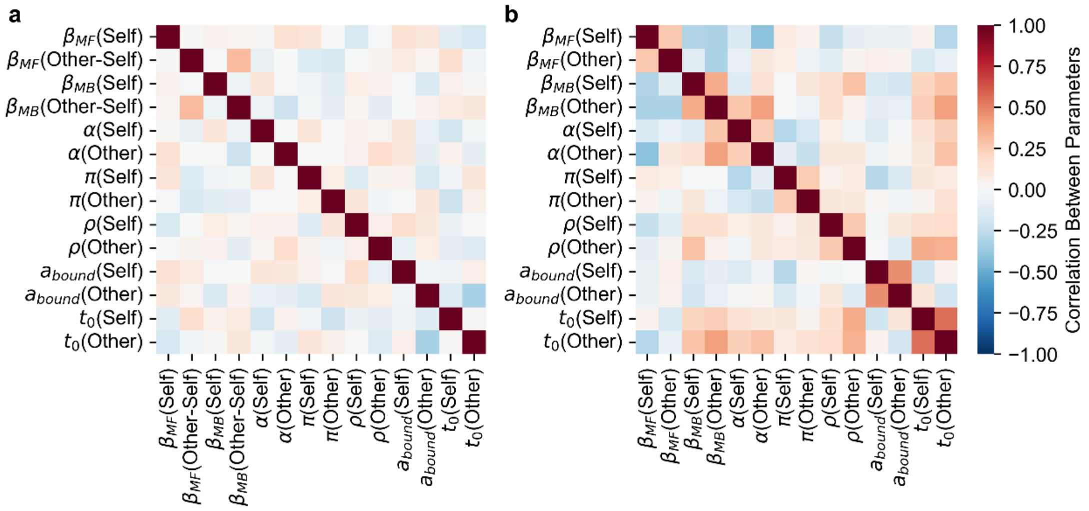

Supplementary Fig. 1: Intercorrelations between estimated parameters. (a) Intercorrelations between recovered parameters estimated from the simulated data. The absolute values of pairwise correlations < 0.33. (b) Intercorrelations between parameters estimated from the empirical data, capturing individual differences in model parameters. For example, we observed high correlations between the same parameters across two conditions, reflecting the within-subject nature of these parameters, such as decision boundaries in Self and Other conditions (r = 0.46) and non-decision time in Self and Other conditions (r = 0.56). These high correlations indicated individual differences in decision cautiousness and time required to process decisional information, respectively. For other correlations, the absolute values < 0.42.

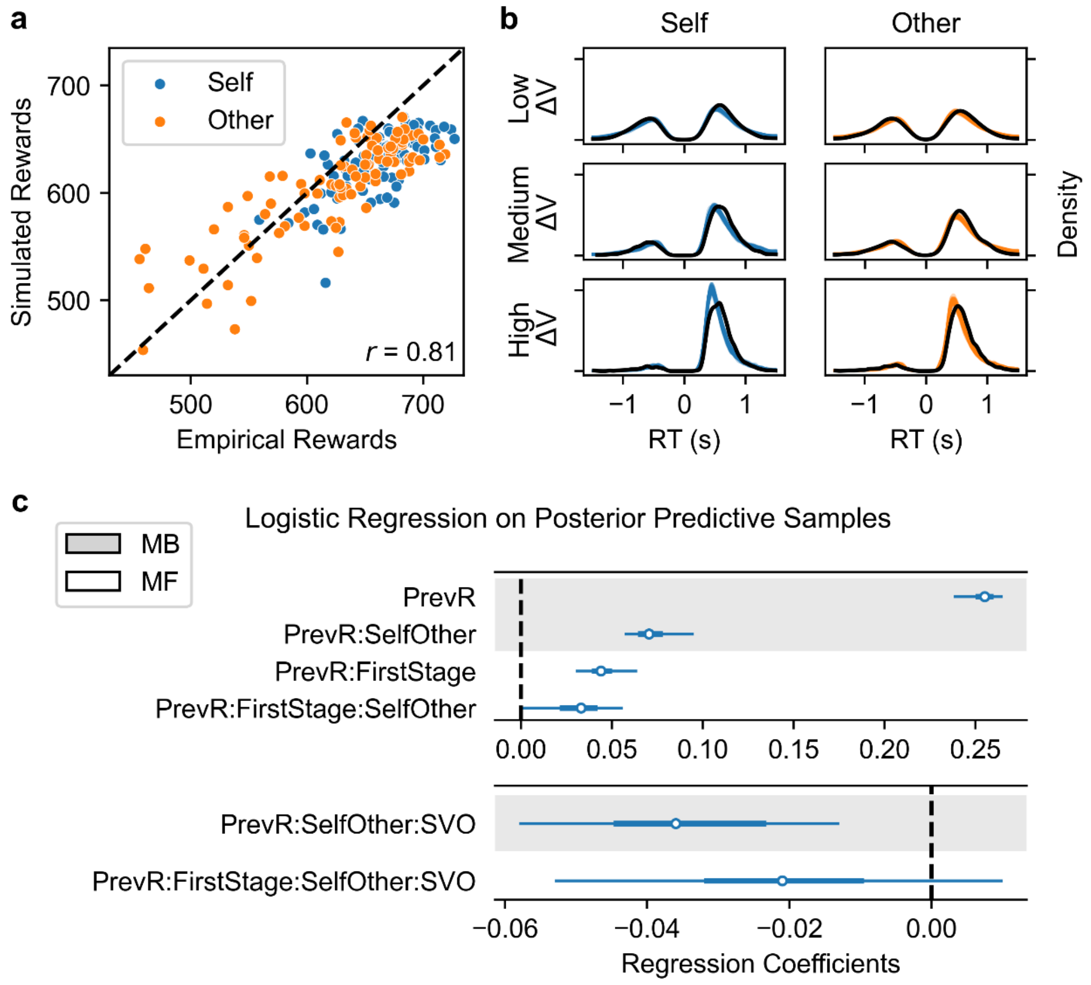

Supplementary Fig. 2: Posterior predictive check. (a) The simulated rewards captured empirically observed individual differences in the rewards obtained. (b) Reaction time (RT) distributions as a function of Self/Other conditions and value differences between two options. Notably, the value here refers to the sum of model-based, model-free values, choice stickiness and response stickiness. The positive RTs indicate decision time when choosing the high-valued options. The negative RTs indicate decision time when choosing the low-valued options. The low, medium and high ΔV are determined based on the terciles of absolute value differences between two options. The blue and orange lines represent posterior predictive samples, whereas the black lines are empirical RT distributions. (c) Logistic regression results on 50 posterior

predictive samples, showing the summary statistics of 50 sets of regression coefficients. The thinner lines are 95% HDI, the thicker lines are interquartile range (IQR), and the empty circles are mean estimates. The dashed lines indicate the reference value (zero) of the regression coefficients. The main effect of the previous outcome was labelled as PrevR. The interaction effect between previous outcome and first-stage state similarity was denoted as PrevR:FirstStage. Regression coefficients related to model-based systems were displayed on the grey background, whereas those related to model-free systems were presented on the white background.

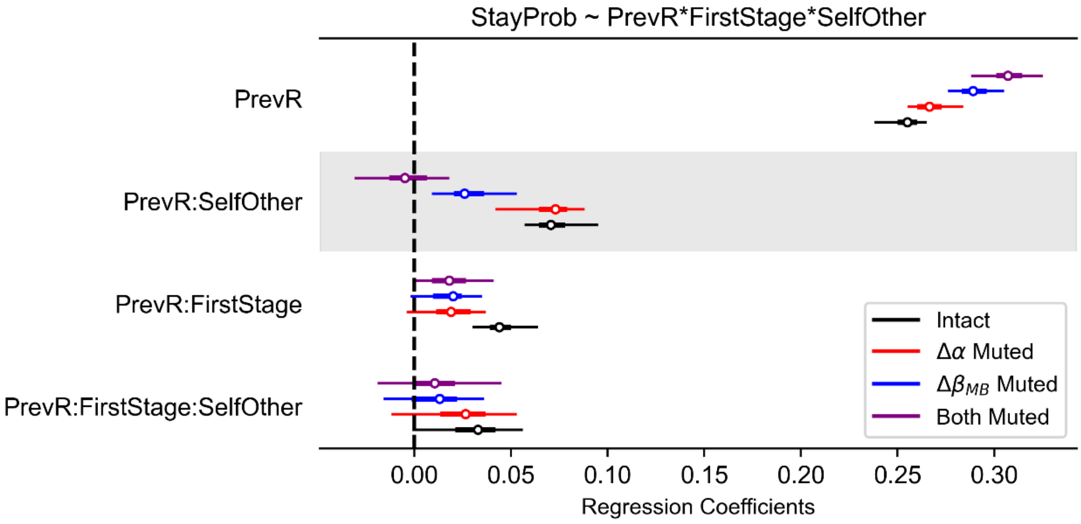

Supplementary Fig. 3: Simulation study results. Four different parameter sets were used to generate simulated data sets. The figure shows the logistic regression results on these simulated data. Black lines represent the original parameter set having intact self-other differences. Red lines represent the parameter set whose self-other differences in model-free learning rates were muted. Blue lines represent the parameter set whose model-based weights were set to the same across the two conditions. Purple lines represent the parameter set whose model-based weights and model-free learning rate were equalised across two conditions. The thinner lines represent the 95% HDI, the thicker lines represent the interquartile range (IQR), and the empty circles represent the mean estimates. The dashed lines point out the reference value (zero). The regression coefficients of interest, i.e., PrevR:SelfOther, are highlighted with a grey background.

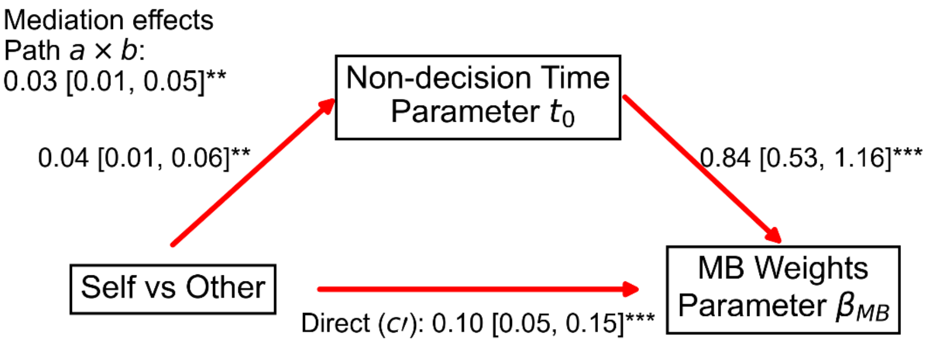

Supplementary Fig. 4: The mediation analysis result when the individual variability in SVO scores was included as a control variable. We show the coefficients and 95% CI of each path. Significant path coefficients are marked with stars and presented as red arrows.
